# Why Selenocysteine Is Unique?

**DOI:** 10.3389/fmolb.2020.00002

**Published:** 2020-01-22

**Authors:** Vitor Hugo Balasco Serrão, Jessica Fernandes Scortecci

**Affiliations:** ^1^Laboratory Medicine and Pathobiology, University of Toronto, Toronto, ON, Canada; ^2^Department of Biochemistry and Molecular Biology, University of British Columbia, Vancouver, BC, Canada

**Keywords:** selenocysteine, genetic code expansion, protein biosynthesis, tRNA specificity, elongation factor

## Introduction

The genetic code was defined as degenerated based on Crick's frozen accident theory (Crick, 1968). Amino acids present different incorporation frequencies; however, specific codons must be responsible for the proper delivery and incorporation into the nascent polypeptide chain (Woese et al., 2000). The genetic code degeneration is not static, and its dynamism can be identified by non-usual amino acids. One example is the selenocysteine (Sec, U), denominated as twenty-first amino acid discovered in the early'80s, which is co-traditionally incorporated in the frame using a UGA-codon recognition (Diamond et al., 1981). This codon is canonically identified as a stop-codon; however, the cell machinery found a way to change this meaning into an amino acid incorporation position. Interestingly, UAG-codon, another stop-codon, can be recognized as Sec or pyrrolysine (Pyl, O), another non-canonical co-traditionally incorporated amino acid (Srinivasan et al., 2002).

Over the years, the molecular mechanism that allows the UGA-misinterpretation for Sec incorporation was identified as being dependent on a specific mRNA element namely SECIS (SElenoCysteine Incorporation Sequence). This unique sequence in the mRNA folds in a hairpin conformation downstream, in Bacteria, or at the 3′ untranslated region–3′UTR in Archaea and Eukarya, changing the interpretation from the canonical UGA-stop codon to UGA-Sec incorporation (Su et al., 2005). The whole Sec incorporation mechanism is fully described in bacteria, where the SECIS element is unfolded by the ribosome movement during the translation process in presence of a specific elongation factor for Sec incorporation (SelB or EFSec) (Fischer et al., 2016). However, the mechanism of SECIS positioning on the top of the UGA-codon position during Sec incorporation in Archaea and Eukarya is still unknown.

Another particularity involving Sec is the Sec-specific tRNA. The tRNA^Sec^ is present in most of the organisms and have a different “8+5” (Bacteria) or “9+4” (Archaea/Eukarya) cloverleaf conformation in comparison with the traditional “7+5” acceptor/TψC-loop fold (Serrão et al., 2018). Obviously, the UCA-anticodon is another key for its specificity, which also includes the longest variable-arm. Each specific characteristic is critical for the tRNA maturation and loading during the Sec biosynthesis pathway.

Initially, the tRNA^Sec^ is not loaded with a Sec amino acid, but with serine (Ser, S) by the seryl-tRNA synthetase (SerRS), resulting in an intermediate Ser-tRNA^[Ser]Sec^. Usually, amino-acyl tRNA synthetases are highly specific enzymes that recognize unique tRNAs molecules to charge with their specific amino acids. However, SerRS is a class II amino-acyl tRNA synthetase that has a particular recognition against the acceptor and the long variable arm from the Ser and/or Sec tRNA, which provides a non-specificity against the anti-codon (Schimmel and Soll, 1979).

The intermediate Ser-tRNA^[Ser]Sec^ is delivered to selenocysteine synthase–SelA in Bacteria–or phosphoseryl-tRNA kinase–PSTK in Archaea/Eukarya–for Ser/Sec conversion. In bacteria, the homodecameric SelA, a pyridoxal-5′-phosphate (PLP) dependence enzyme, is responsible for this conversion forming a ternary 1.3 MDa complex machinery (Silva et al., 2015). This transient complex is assembled by the interaction between SelA.Ser-tRNA^[Ser]Sec^ binary complex and an enzyme named selenophosphate synthetase (SelD), responsible for delivering the selenium donor–selenophosphate–and provides the catalytic pocket to obtain the mature Sec-tRNA^Sec^ (Silva et al., 2015). Selenium compounds are cytotoxic, which may require a dedicated mechanism to avoid cell death. An alternative to keep low toxicity levels is recycling selenium compound into the biological and useful source as selenophosphate. This conversion involves a non-Sec pathway-specific enzyme–selenocysteine lyase (CsdB)–that drives the Sec recycling to selenide, which is highly toxic for the cells. Results suggested that CsdB.SelD interacts to protect the environment and catalyzes the selenium phosphorylation regardless of the organisms (Itoh et al., 2009).

On another hand, in Archaea and Eukarya the Sec biosynthesis is different and divided into two distinguished steps. The initial Ser residue present in the Ser-tRNA^[Ser]Sec^ is phosphorylated by PSTK, resulting in an intermediate o-phosposeryl-(Sep)-tRNA^[Sep]Sec^. Following the conversion, the o-phosphoseryl-tRNA^Sec^ selenium transferase (SepSecS–a PLP-dependent enzyme) performs the Sep/Sec conversion, resulting in the mature Sec-tRNA^Sec^ (Liu et al., 2014). The biological selenium compound is delivered as mentioned for bacteria.

Another main difference between Sec and other amino acids is the incorporation process. Mature Sec-tRNA^Sec^ is recognized and specifically delivered into the ribosomal machinery by unique elongation factors (SelB or EFSec), which have the capability not only to recognize the traditional molecules for amino acids incorporation, i.e., L30 ribosomal subunit and tRNA, but also to recognize the SECIS and/or SECIS-binding proteins (SBPs) (Fletcher et al., 2001). SBPs are proteins founded in Eukarya that identify SECIS elements on 3′-UTR and help its interaction with eEFSec for Sec incorporation (Fletcher et al., 2001). In bacteria, SelB is capable to identify all three elements (ribosome, mature tRNA^Sec^ and SECIS element). This mechanism is the key that allows the UGA-misinterpretation and introduces Sec as part of the nascent polypeptide. This mechanism inspired researchers in the new efforts to understand and use this genetic code expansion to improve protein engineering and synthetic biology (Miller et al., 2015). Mutations and chimeras made possible non-specific incorporation of amino acids into different codons, i.e., perform the incorporation of canonical amino acids at the UGA-codon by using elongation factors (EFTu) fused with SelB-Cterminal domain, responsible for SECIS recognition, as an example (Soll, 2015). Further improvements in synthetic biology are ongoing and the results will inspire, and grounds break this field in the nearly future.

Moreover, the full understanding of this incorporation mechanism is currently helping the protein engineering process and synthetic biology, allowing a “new expansion” of the genetic code by manipulating the incorporation of the amino acids (Mukai et al., 2017).

Sec is one example of this expansion that nature made to increase the variability in the proteins, function and also prevents cell toxicity. Fully understanding of this mechanism may help us to understand protein evolution processes and macromolecular interactions that will allow us to design new methods and ways to expand the possibilities into synthetic biology.

## Discussion

The importance and uniqueness of the Sec incorporation elongation factor are highlighted when the complex system was fully described in 30 years of extensive research. Aspects like how it can regulate selenoproteins expression under oxidative stress or how to pursue the UGA-stop misinterpretation are fascinating to understand and expand the genetic code. New ways to rewrite or interpreting the same information will expand the frontiers of the genetic code, not only by increasing the diversity and possibilities in protein engineering, but also creating new insights to codify specific enzymes and proteins. The whole Sec biosynthesis pathway is unique, however, the EF-UGA recognition is the main reason that Sec is different. EF usually recognizes the incorporation machinery at the proper position for the amino acid incorporation.

Moreover, this mechanism can be used to answer questions about the incorporation process efficiency, accuracy, and conservation among different species. Sec incorporation is different mainly because of SelB, the special EF that allows the UGA-Sec recognition based on an mRNA element. Evolutionary, Sec-EF changed to recognize another partner that helps to properly guide the Sec-tRNA^Sec^ into the ribosomal cavity, making this process more efficient.

As previously mentioned, researchers already started to understand and use the Sec machinery to create chimeras that allow us to change the codon's recognition. Further studies in EFs and its interactions using SelB/eEFSec as the example may guide us for new insights in the new genetic expansion era.

## Author Contributions

All authors listed have made a substantial, direct and intellectual contribution to the work, and approved it for publication.

### Conflict of Interest

The authors declare that the research was conducted in the absence of any commercial or financial relationships that could be construed as a potential conflict of interest.
